# Customized ViNeRS Method for Video Neuro-Advertising of Green Housing

**DOI:** 10.3390/ijerph17072244

**Published:** 2020-03-27

**Authors:** Arturas Kaklauskas, Edmundas Kazimieras Zavadskas, Bjoern Schuller, Natalija Lepkova, Gintautas Dzemyda, Jurate Sliogeriene, Olga Kurasova

**Affiliations:** 1 Department of Construction Management and Real Estate, Faculty of Civil Engineering, Vilnius Gediminas Technical University, Sauletekio av. 11, LT-10223 Vilnius, Lithuania; natalija.lepkova@vgtu.lt (N.L.); jurate.sliogeriene@vgtu.lt (J.S.); 2 Department of Computing, Faculty of Engineering, Imperial College London, South Kensington Campus, London SW7 2BU, UK; bjoern.schuller@imperial.ac.uk; 3 Cognitive Computing Group, Institute of Data Science and Digital Technologies, Vilnius University, Universiteto st. 3, LT-01513 Vilnius, Lithuania; gintautas.dzemyda@mii.vu.lt (G.D.); olga.kurasova@mii.vu.lt (O.K.)

**Keywords:** green housing, neuro decision matrix, neuro correlation matrix, video neuro-advertising, COPRAS and ViNeRS Methods, multivariate design and multiple criteria analysis

## Abstract

The implementation of advertising for green housing usually involves consideration of individual differences among potential buyers, their desires for residential unit features as well as location impacts on a selected property. Much more rarely, there is consideration of the arousal and valence, affective behavior, emotional, and physiological states of possible buyers of green housing (AVABEPS) while they review the advertising. Yet, no integrated consideration of all these factors has been undertaken to date. The objective of this study was to consider, in an integrated manner, the AVABEPS, individual differences, and location impacts on property and desired residential unit features. During this research, the applications for the above data involved neuromarketing and multicriteria examination of video advertisements for diverse client segments by applying neuro decision tables. All of this can be performed by employing the method for planning and analyzing and by multiple criteria and customized video neuro-advertising green-housing variants (hereafter abbreviated as the ViNeRS Method), which the authors of this article have developed and present herein. The developed ViNeRS Method permits a compilation of as many as millions of alternative advertising variants. During the time of the ViNeRS project, we accumulated more than 350 million depersonalized AVABEPS data. The strong and average correlations determined in this research (over 35,000) and data examination by IBM SPSS tool support demonstrate the need to use AVABEPS in neuromarketing and neuro decision tables. The obtained dependencies constituted the basis for calculating and graphically submitting the ViNeRS circumplex model of affect, which the authors of this article developed. This model is similar to Russell’s well-known earlier circumplex model of affect. Real case studies with their related contextual conditions presented in this manuscript show a practical application of the ViNeRS Method.

## 1. Introduction

Soon it will be fifty years since researchers such as Fisk [1] and Henion and Kinnear [2] integrated ecological questions into the marketing approach and presented concepts such as ecological marketing. Notwithstanding the ubiquity of green/environmental research in marketing works, rarely did such experiential investigations lead companies to incorporate and operationalize green marketing in their normal professional activities [3].

Green marketing focuses on the arousal and valence, affective behavior, emotional, and physiological states of possible buyers of green housing (AVABEPS). Based on such AVABEPS (e.g., happiness), buyer shopping needs and priorities can be identified. A brief overview of several studies in this area follows. Using housing satisfaction as explanatory variables, Zhang et al. [4] believe that housing satisfaction and home ownership significantly contribute to overall happiness. Functional and emotional value, for many, is likely the dominant value dimension that, among personal health causes, consumers seek in social marketing. Emotional worth is linked to numerous affective states that can be either positive or negative [5]. When personal health causes are considered, this can mean either the suppression of negative affective states or the promotion of positive affective states [6]. The occupants of green buildings, in general, were more satisfied than those in conventional buildings [7]. As the geographical detector model shows, overall satisfaction with urban livability is significantly and positively affected by all six dimensions of urban livability, with convenient transportation, the natural environment, and environmental health having the biggest impact [8].

The definition of green marketing positioning is coined to address a firm’s holistic positioning relative to the natural environment [9]. It indicates the tailoring of a circular economy positioning to keep the value of resources, materials, and products for as long as possible [10]. A life cycle assessment (LCA) can avoid a narrow viewpoint on sustainability concerns [11]. In the late 1980s, new instruments were invented such as LCA, with which ecological considerations could be introduced into marketing decisions [12].

Papadas et al. [9] propose that tactical actions (i.e., the use of resources, materials and products, and green pricing guidelines) suggest elasticity to executives for correcting their green marketing plan according to micro- and macro-environmental variations. It is possible to accentuate the advantages to residents of green buildings (material, water, and energy efficiency; reusable, recycled, and low-impact structure materials; waste lessening; low-carbon machineries; inhabitant health and inside environmental quality; and renewable energy) and their environments (air and water pollution reduction and human health, green architecture, green-built environment), depending on the micro-, meso- and macro-environment during the time of the advertising. It is also possible to point out the environmental reputation, values, culture, and behavior of the companies that constructed these buildings. The holistic advertising of the aforementioned green buildings and their environments in time and space would result in a more synergetic marketing effect. To achieve such a purpose, the effort was to establish the needs for green housing at the locales under analysis.

An empirical study on tourist segmentation that Bigné and Andreu [13] presented used the consumption emotions by dimensions of pleasure and arousal as its basis. The enjoyment of leisure and tourism services evokes such consumption emotions. That emotion is suitable as a segmentation variable was supported by these obtained results. Greater levels of pleasure and arousal indicated experiences of increased satisfaction. Furthermore, these also indicated increases in favorable behavioral intentions, which meant greater loyalty and willingness to pay more [13]. The emotional profiles and segments of tourists along with their post-consumption evaluations of satisfaction related to their intentions to recommend, as per Hosany and Prayag [14]. Del Chiappa et al. [15] have studied emotions as a potential variable for segmenting museum audiences. Positive emotions were reported for the audience segment that perceived the museum as being a unique attraction, and this segment also reported greater satisfaction with their museum experience [15].

Emotion can encourage good decision-making, according to Nobel Laureate Simon [16], who has analyzed the role of emotions in decision-making and concluded a lack of any intrinsic conflict between rationality and emotions. Furthermore, there is Pham [17], who proposes using fleeting feelings as credible sources of information when it comes to consumption decisions. The somatic marker hypothesis states that there are bodily signs relevant to experiencing a positive outcome that makes people feel happy and motivates them to continue seeking the same sort of behavior. Negative perception of a bodily sign will result in feelings of sadness, which initiates an internal warning to avoid acting in some certain way. The specific situation thus triggers a certain bodily sign and serves to guide behavior, because its basis relates to some past experience. This process reinforces choice as offering a more advantageous outcome, which could be considered adaptive [18]. The 80/20 rule for decision-making about purchases is popular worldwide. This rule proposes that purchasing decisions are based 80% on emotions and 20% on logic. The authors of this article have also engaged in studies indicating the strong role of emotions while analyzing decision-making alternatives.

It is usual to advertise green housing taking into account individual differences, location impacts on property variables, and the looked-for qualities of a dwelling [4,7]. But looking at variables such as the emotional, affective, and physiological reactions, and arousal and valence [19,20,21] that characterize a potential buyer is less common. Real estate and construction sector companies have simply ignored these factors in their advertising campaigns and never considered making an integrated multivariant design of video ad alternatives. This research is an attempt to fill this knowledge gap by examining all the factors mentioned above. For that purpose, the ViNeRS Method developed by the authors is used. This integrated study could highlight interrelated aspects that have never been analyzed before.

This article begins with the introduction followed by Section 2 on methodology, Section 3, which contains case studies for illustration, Section 4 on the correlational analysis between emotions and a built environmental state and closes with the conclusions.

## 2. Method

**Figure 1:**
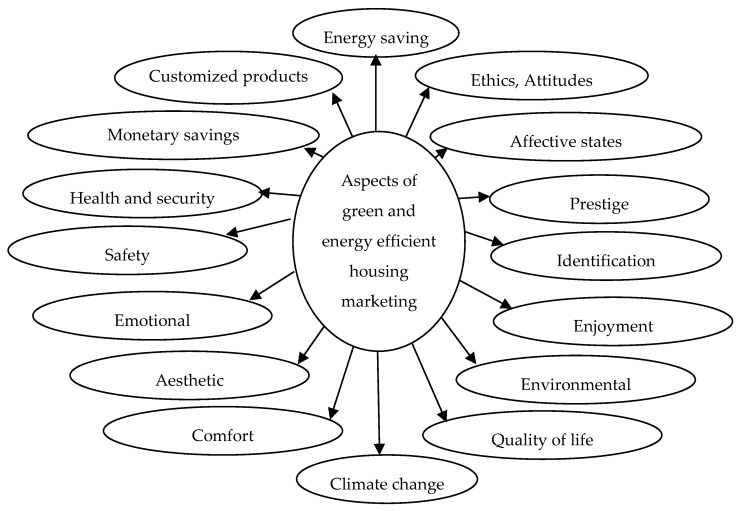
Quantitative and qualitative aspects of green and energy efficient marketing for analyzing the life cycle of a built environment.

The initial discussion will be pertinent to the life process of a green and energy-efficient building and the quantitative and qualitative aspects of its marketing. A multisensory, green, and energy-efficient housing neuromarketing method was developed to integrate various aspects of the green marketing process, such as energy, the environment, health, economics, laws/regulations, innovation, the microclimate, and social, cultural, ethical, psychological, religious, ethnic, and other related matters with the life cycle of a built environment (see Figure 1). A number of researchers have analyzed various marketing variables. Jamal and Sharifuddin [22], for instance, analyzed marketing literature and concluded that the concept of perceived value is well established in that context and has been used to examine variables that play a role in future purchase decisions, as well as in the use of services and products.

Dangelico and Vocalelli [23] believe that the qualities that make a product green must be valuable and perceivable. When strength-related attributes (e.g., being long lasting) of a product are valued, sustainability could be an advantage, because, compared with other products, green products are often seen as healthier, safer, and gentler [24].

Functional and emotional values, for many, are likely the dominant value dimensions that consumers seek in social marketing for personal health causes. Emotional value relates to various affective states that can be either positive or negative [5]. When personal health causes are considered, this can mean either the suppression of negative affective states or the promotion of positive affective states [6].

Hedonic value and utilitarian value are important aspects of business and retail strategies. Other crucial concerns in management and marketing are customer satisfaction and brand loyalty [25]. The nature and theory of marketing experience, however, suggest that, specifically in a shopping context, the link between satisfaction and hedonic value should be stronger than that between satisfaction and utilitarian value [26].

Marketers promoting a product with mainly hedonic characteristics (enjoyment, identification, prestige, and general positive experiences) should focus on collaborating with marketers who can serve an appealing leadership function [27].

Figure 1 presents the general layout of the multisensory, green, and energy-efficient housing neuromarketing method, which the authors of this research developed.

Occupants in green buildings were generally more satisfied than those in conventional buildings [7]. As the geographical detector model shows, all six dimensions of urban livability, including convenient transportation, a natural environment, and good environmental health, significantly and positively have the greatest impact on overall satisfaction with urban livability [8].

ViNeRS is a customization method. Mass customization means that each individual customer gets what he or she needs, but mass production efficiency can still be ensured [28]. As defined by Pine [29], mass customization is the efficient production of individually customized offerings in high volumes and at a low cost.

**Figure 2:**
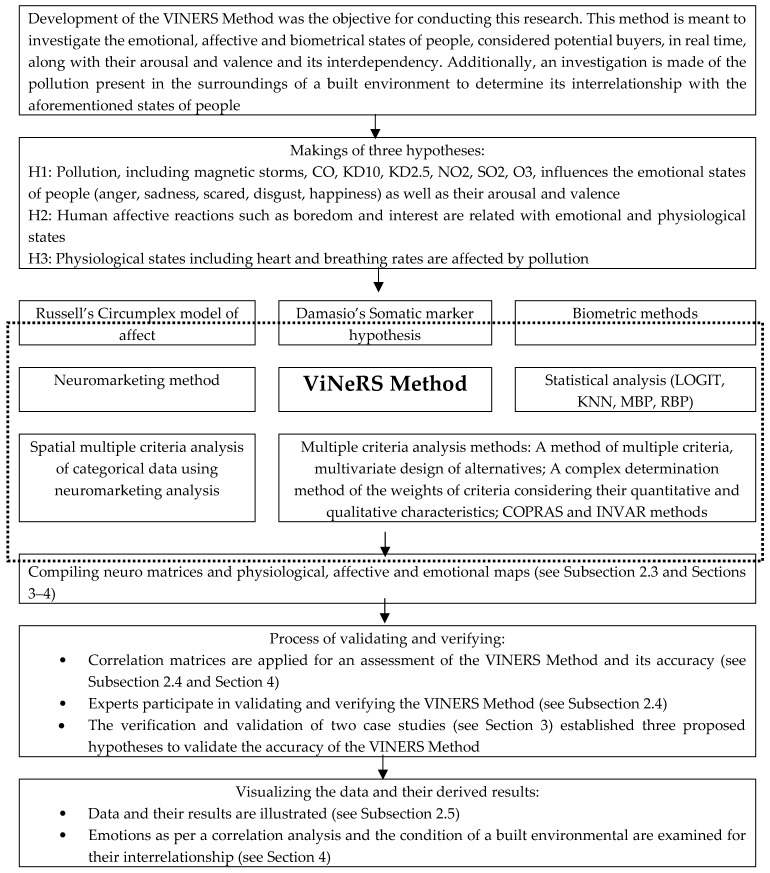
Main phases of the Video Neuroadvertising Recommender System (ViNeRS) Method.

The multivariant planning and the multiple criteria analysis of video neuro-advertising variants (hereafter referred to as the ViNeRS Method) goes through seven stages. The key phases of the ViNeRS Method appear in Figure 2.

Brief descriptions of these phases follow.

This article aims to showcase the offers to users made possible by the proposed ViNeRS Method.

### 2.1. The Research Problem and Hypotheses

Advertising for green housing units must integrate the looked-for qualities of a dwelling and the respective location impacts on the proposed property, with consideration of how the arousal and valence, affective behavior, emotional, and physiological states, affect the attitudes of possible buyers of green housing (AVABEPS), and individual differences of the potential buyer. Usually, advertising for green housing is executed in consideration of the individual differences, the looked-for qualities of a dwelling, and the location impacts on the property [4,7]. However, consideration of the emotional, affective and physiological reactions as well as arousal and valence [20,21] of a potential buyer occurs markedly less often. Despite this, the advertising campaigns of real estate companies simply do not consider these factors and the multivariant design of ad alternatives in an integrated manner at all. This research aims to fill this gap by examining all the above factors and the multivariant planning of variants in an integrated study. This research could support the highlighting of interrelated aspects, which have not been analyzed previously.

Development of the ViNeRS Method was the objective in conducting this research. This method is meant to investigate the emotional, affective, and biometrical states of people, considered potential buyers, in real time, along with their arousal and valence and its interdependency. Additionally, an investigation is made of the pollution present in the surroundings of a built environment to determine its interrelationship with the aforementioned states of people.

The combined experiences of the authors of this article, together with intuition and the analysis of scholarly literature, contributed to formulating three hypotheses (see Figure 2) for this research.

The application of the ViNeRS Method, which the authors of this article developed, substantiated these hypotheses.

### 2.2. ViNeRS Method

The ViNeRS Method consists of a methodological integration of the Somatic Marker Hypothesis proposed by Damasio [18], Russell’s circumplex model of affect [30], and other components, including biometric methods [31]; use of neuro-marketing analysis methods with categorical data for a spatial multiple criteria analysis employing, for example, AVABEPS maps [32]; LOGIT model, k-nearest neighbors algorithm (KNN), Marquardt backpropagation (MBP) algorithm, and recurrent backpropagation (RBP) statistical analyses, neuromarketing methods, application of multiple criteria, multivariant design of alternatives [33,34], and multiple criteria analysis methods for establishing criteria weights [33,34]; and the COPRAS [33,34,35] and the INVAR methods [36]. The multiple criteria, multivariant design and multiple criteria analysis methods itemized here include a detailed summary along with a number of examples of practices previously described [33,34,35]. There is also a detailed overview of the COPRAS technique, which also includes examples [33,34,35].

### 2.3. Compiling Neuro Matrices and Developing AVABEPS Maps

Four quantitative and qualitative strata of data were accumulated in this stage of the research. The work proceeded by a systematic study of these data.

The authors of this contribution believe that only a neuro decision matrix (which includes criteria, their values and weights) can ensure that the apartment for sale and its surroundings can be described in detail, and that the arousal and valence, affective behavior, emotional, and physiological states, affect the attitudes of possible buyers of green housing (AVABEPS) as well as how individual differences in people present in a public space can be tracked to achieve a more systematic analysis of the neuromarketing process related to an apartment. The neuro decision matrix is a tool that offers real-time mapping of the AVABEPS of people present in a public space. The matrix also offers integrated analysis of expert judgement results retrieved from various databases (location impacts on property, individual differences, features of an apartment).

The result of our research is unique, with integrated data related to the apartment for sale, its surroundings and potential buyers (arousal and valence, emotional, affective and physiological reactions, and individual differences). In the development of a neuro decision matrix, a key stage is determining what set of criteria will describe the alternative video advertisements that promote apartments, which units of measurement will be used, and what the weights and values will be. The quantitative and qualitative data of these alternative video advertisements are an important contributor to neuromarketing efficiency because they comprehensively describe the alternatives considered in the research.

The outcomes from the examination of ad options are delivered in the neuro decision matrix. The columns describe the ad variants under analysis, and the rows describe the data on the criteria that thoroughly define the video alternatives for the green housing unit under deliberation. A more detailed description of the neuro decision matrix appears in the case study write-up.

Equipment, which consisted of remote, biometric analysis devices (Respiration Sensor X4M200, the H.264 Indoor Mini Dome IP Camera and FaceReader 7.1) establish the affective attitudes (interest, boredom), emotional states (anger, sadness, scared, disgust, happiness), valence, arousal, and physiological states (breathing and heart rates). Measurements were taken every second. Examinations of passersby regarding their affective attitudes and emotional, affective, and physiological states took place at four intersections in Vilnius under analysis, from November 2017 until February 2020. Over 350 million pieces of data were collected during this period.

The accumulation of six layers of data validated these three hypotheses (see Figure 2) and aided the completion of this research:

- Potential buyers’ individual differences: location impacts on property and desired residential unit features—data gained from the Lithuanian Department of Statistics (X_1_–X_6_).
- Green housing unit attributes (material, water, and energy efficiency; reusable, recycled, and low-impact structural materials; waste lessening; low-carbon machineries; inhabitants health and inside environmental quality; renewable energy)—data gained from the Lithuanian Department of Statistics (X_8_, X_9_), the Environmental Protection Agency (X_11_), real estate brokers and experts in the field (X_7_, X_10_, X_12_, X_13_).
- Location impacts (urban quality and infrastructure (X_14_) and green spaces (X_15_))—data gained from the real estate brokers and experts in the field.
- AVABEPS data measured by the respiration sensor X4M200 and FaceReader 7.1 (X_16_–X_19_).

The fourth presented layer of data served as the basis for compiling maps locating the emotional, affective and physiological reactions of potential buyers of residential units.

This marketing segmentation involved the division of a wide-ranging customer base containing existing and potential consumers into subgroups of customer segments grounded on the above-named features. The general goal of this segmentation was to recognize the subgroups of customers that are the most likely purchasers of green housing units or the greatest money-making customers. Consequently, these possible buyers can be targeted according to their demographics, behavior, or any other added segments of significance. Marketing segmentation accepts that diverse marketing segments need various advertising alternatives with different dwelling variants (prices, qualities, location impacts, and other variables). The marketing segmentation goal here is to generate profiles of the main potential dwelling buyers.

Assuming, for example, that passers-by of a certain age group in a district under deliberation feel better (i.e., display more indicators of positive emotions such as happiness) and show fewer indicators of negative emotions (e.g., angry, sad) compared to other age groups with analogous indicators; then, the weight of importance for this age group is greater. The converse is also true. This corresponds with results gained by other researchers [37,38,39].

In the future, additional criteria may be added that will allow users to choose manually the location impacts, individual differences, or desired features of a dwelling along with other criteria for consideration when developing video ad alternatives. When the multiple criteria analysis of suitable video ad alternatives aimed at some specific segment is performed, potential buyers are shown video advertisements of dwellings that are best matched to their needs. Once a decision is made regarding the advertising aimed at a segment of specific, potential green housing buyers, the pool of video advertisements is reduced, ideally to include only those of interest to a potential buyer of a green housing unit. Nonetheless, in reality, only a fraction of the ads in that limited pool will actually appeal to a buyer.

Compilations of biometric, physiological and emotional maps based on the developed neuro decision tables [32] are accomplished.

### 2.4. Validating and Verifying for Applying the Method in Practice

Initially, the special, custom-made ViNeRS Method was verified for an evaluation of its accuracy. The application of correlation matrices is the means to prove that the results obtained by the ViNeRS Method are indeed relevant to a situation in reality. The principal goal of this phase was to define any prevailing associations between the AVABEPS emotional states (anger, sadness, scared, disgust, happiness), arousal and valence, affective reactions, and physiological states (heart and breathing rates) of the passers-by in question, and built-environment pollution (SO_2_, NO_2_, CO, KD_2.5_, KD_10_, O_3_, and magnetic storms). The correlations determined between the variables (the neuro correlation matrix) support the necessity to use AVABEPS variables in neuro decision tables and neuromarketing.

This article presents realistic, in-depth and detailed descriptive case studies in a naturalized setting to serve as a practical demonstration of the ViNeRS Method. The overall objective of these case studies is to analyze a specific case by applying the ViNeRS Method for a better understanding of the developed technique.

Validation proved to be the means for establishing the accuracy of the customised ViNeRS Method. A pilot experiment on the practical application of the ViNeRS Method was performed in order to evaluate its efficiency and usability so as to improve the method prior to introducing it into real estate brokering practice. The testing of the ViNeRS Method employed the black-box testing method. The tester was provided with information about the results gained by the ViNeRS Method together with the corresponding data.

There were 18 experts (residents, real estate brokers, and potential real estate buyers) who checked the ViNeRS Method to make sure it was meeting stakeholder expectations. All their reviews, assessing how well stakeholder expectations were met, were presented as a report. This report also included recommended improvements, citations from existing best practices that analyze human emotions and biometric parameters in a public space, and statements on actions taken by the ViNeRS Method developers to improve the method since its last review. The results of this experiment suggest that the analyses of human emotions and the biometric parameters of a public space empower the ViNeRS Method to generate buying opportunities for green housing that meet the needs of different stakeholders more efficiently.

### 2.5. Illustrations of Data and Results

The illustrations of the data and results derived by the ViNeRS Method can be in quantitative forms (tables, graphs, charts, and circles presenting relationships) and in conceptual forms (a written description of the quantitative part). Therefore, it is possible to provide presentations of different neuromarketing alternatives from different perspectives. We present an example of illustrations of data and results in Chapter 4, “Relation of human emotions based on correlation analysis between emotions and built environmental state.”.

## 3. Practical Application

The verification and validation of two case studies established three proposed hypotheses to validate the accuracy of the ViNeRS Method. These hypotheses are the following:

**Hypothesis** **1** **(H1).** *Pollution, including magnetic storms, CO, KD_10_, KD_2.5_, NO_2_, SO_2_, O_3__,_ influences the emotional states of people (anger, sadness, scared, disgust, happiness) as well as their arousal and valence.*

**Hypothesis** **2** **(H2).** *Human affective reactions such as boredom and interest are related with emotional and physiological states.*

**Hypothesis** **3** **(H3).** *Physiological states including heart and breathing rates are affected by pollution.*

### 3.1. Case Study 1: Neuro Correlation Matrix

**Figure 3:**
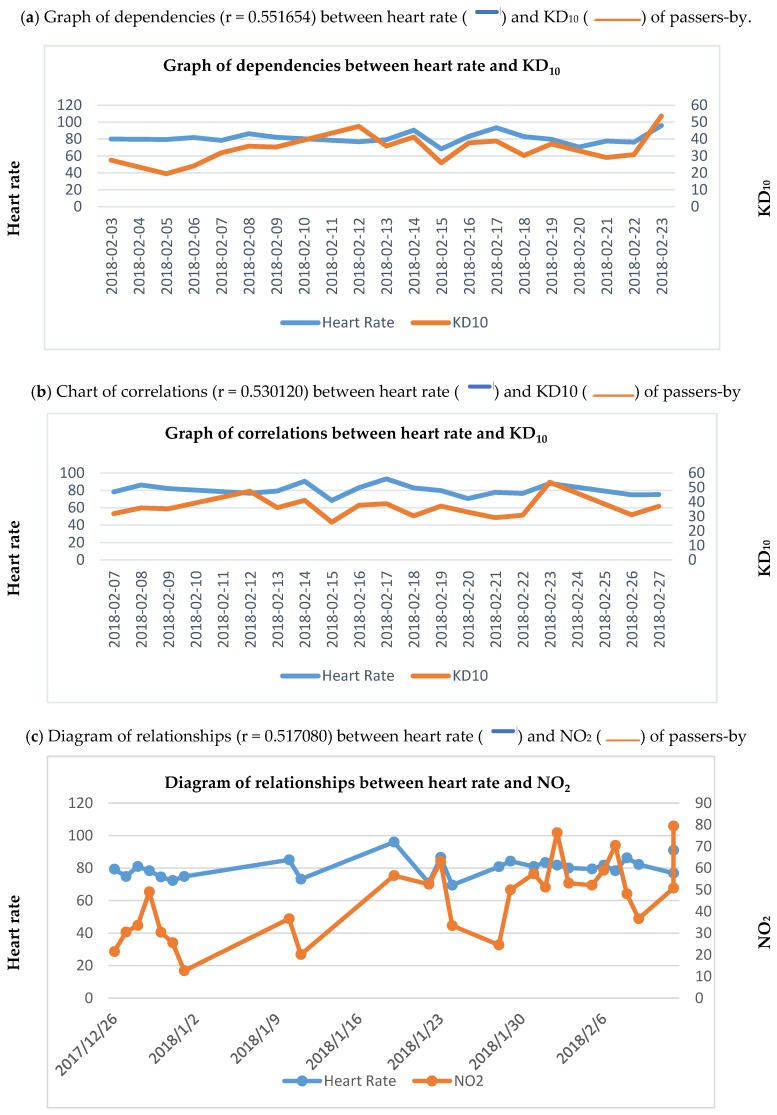

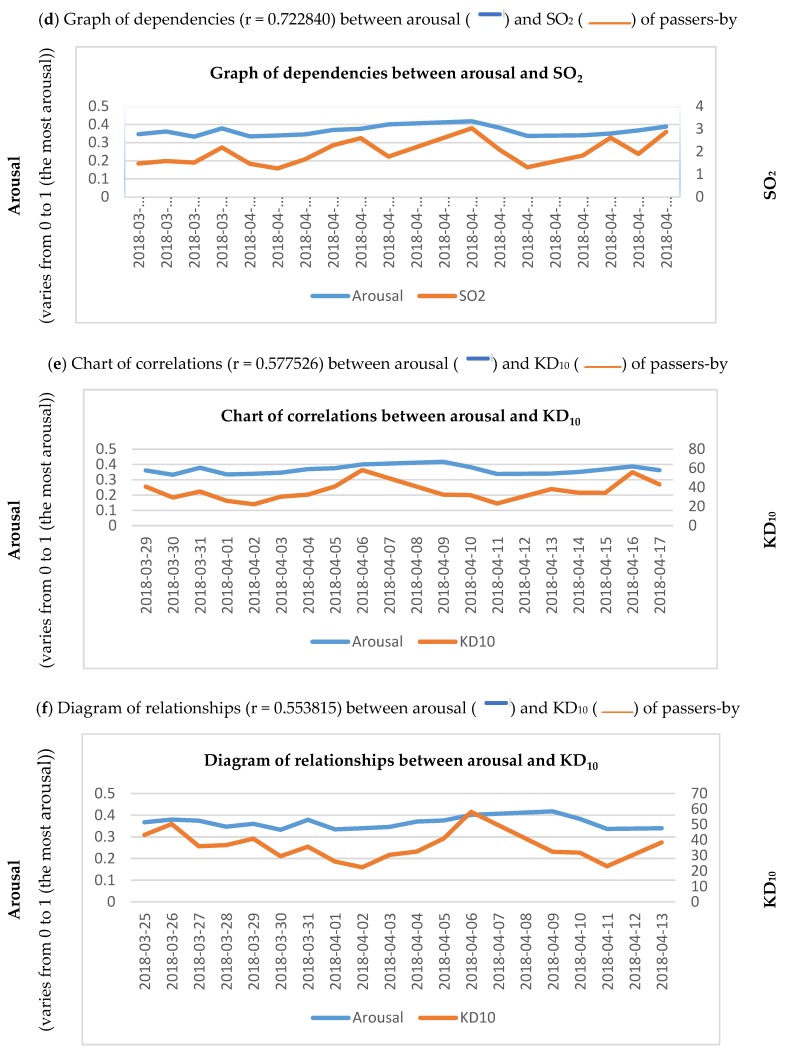

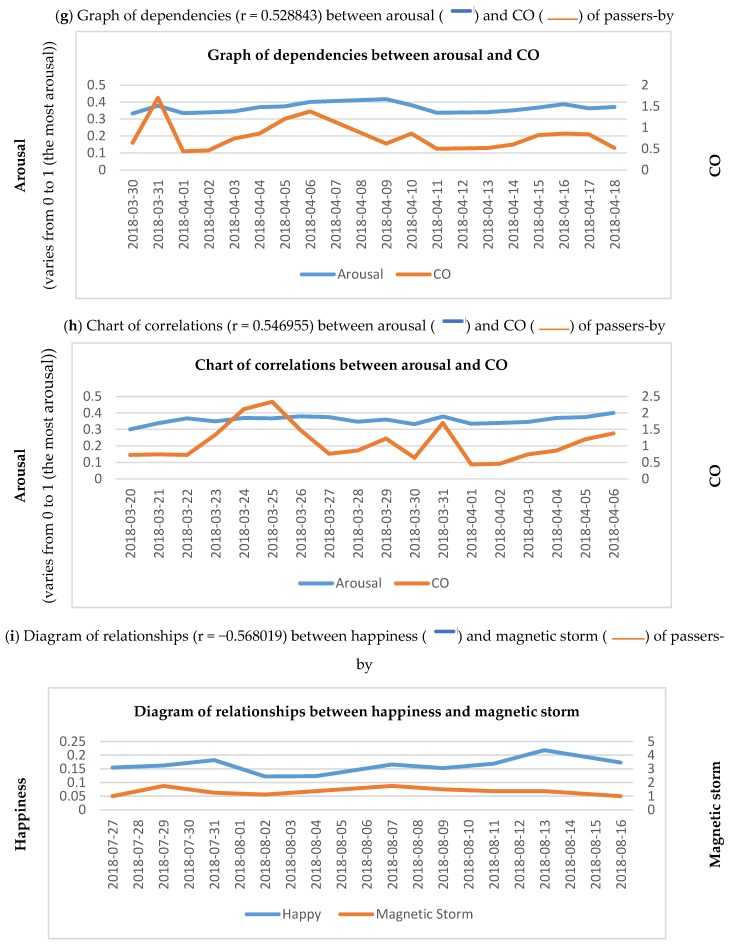

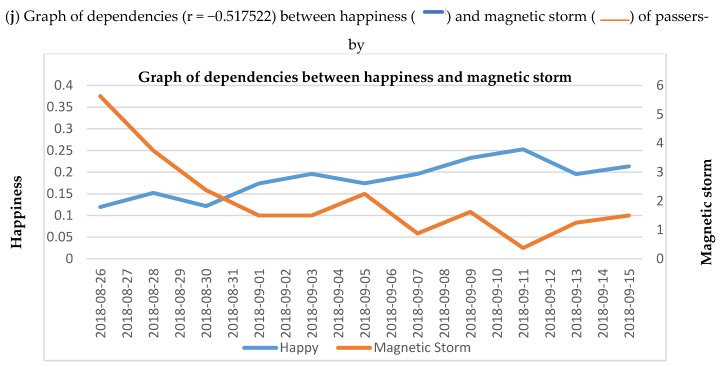
The dependency between average daily (**a**) heart rate and KD_10_, (**b**) heart rate and KD_10_, (**c**) heart rate and NO_2_, (**d**) arousal and SO_2_, (**e**) arousal and KD_10_, (**f**) arousal and KD_10_, (**g**) arousal and CO, (**h**) arousal and CO, (**i**) happiness and magnetic storm, (**j**) happiness and magnetic storm based on the values measured at six Vilnius intersections.

To create a neuro correlation matrix, we need two metrics (variables). Experiments were conducted to gather data for the neuro correlation matrix. For that purpose, we tracked AVABEPS of passers-by; all data was anonymized. The experiments (still ongoing) were launched on 6 November 2017 at seven intersections across Vilnius, Lithuania. Table 1 shows a database snippet with AVABEPS variables of passers-by recorded at four intersections in Vilnius. The AVABEPS layers of data were collected and then processed, integrated, and analyzed. Figure 3 gives several AVABEPS data relationships as an example. With the help of the neuro correlation matrix, over 35,000 average and strong correlations were determined.

The neuro correlation matrix was applied to examine the relationships linking multiple metrics (variables). AVABEPS data were analyzed using IBM SPSS software. Correlational analysis results appear in Table 1.

The accumulation and analysis of more than 350 million data comprised this research, which enabled determinations of more than 35,000 average and strong correlations. The results of our correlational analysis (see Table 2) and the significance of the variable interrelations (see Table 3) support the first hypothesis that “Built environment pollution, including magnetic storms, CO, KD_10_, KD_2.5_, NO_2_, SO_2_, and O_3_, influences the emotional states of people (anger, sadness, scared, disgust, and happiness) as well as their arousal and valence”.

Our findings also show that human affective reactions such as boredom and interest are linked to emotional and physiological states (see Table 4). Global findings also confirm these relationships [40,41,42,43]. The correlational relationships we have determined (see Table 4) and findings by other researchers support our second hypothesis “Human affective reactions such as boredom and interest are related with emotional and physiological states”.

Having examined the correlational relationships, we have determined that physiological states including the heart rate and breathing rate are affected by built environment pollution. Global findings show that environmental pollution affects the heart rate [44,45,46,47] and the breathing rate [48] of people. The correlational relationships we have determined (see Table 5) and findings by other researchers support the third hypothesis that “Physiological states including heart and breathing rates are affected by built environment pollution”.

### 3.2. Case Study 2: Multiple Criteria Analysis of the Segmentation Matrices

Buyers’ individual differences, dwelling characteristics, and location impacts are very significant factors during the selection of an apartment. Previous studies have analyzed individual buyer differences, housing characteristics, and location impacts [49].

Potential buyers were offered views of video advertisements arranged by Vilnius real estate developers featuring various projects offered to the market at the time, ranging from economic to luxury classes. A team was formed consisting of ten experts working as real estate agency brokers, developers, and analysts in Vilnius City. The experts evaluated the advertisement offered and assigned it to an appropriate group of district residents defined by the Department of Statistics under research. There were four age groups divided conditionally: Group I aged 20‒30 years, Group II aged 31‒40 years, Group III aged 41‒60 years and Group IV aged over 60 years.

Group I and II. Ages 20-40 years, the first two groups, have the main purchasing power in Lithuania. The main reason is that ~70% of real estate (RE) acquisitions occur with the participation of a bank, i.e., buyers employ a bank loan. Meanwhile, banks finance specifically these two age groups the most favorably, on their own accord. The ages of these buyers permit them to take a loan with the longest repayment term, and it is likely the incomes of such buyers will only rise. Representatives of these age groups are the most active. Their children still attend kindergartens and schools and participate in various activity groups. Therefore, it is especially important to these buyers to have a location that is accessible as much by automobile as by public transport.

Group III, aged 41–60 years, are buyers who are already buying the second or third housing unit in their life. Their values and customs have already formed, so they are less vulnerable to fashion trends. Their children have grown up, so their priorities become matters such as comfort, a stable neighborhood, and nature. This category of buyers frequently look for real estate for investment purposes and to ensure stable incomes for themselves upon becoming pensioners.

Group IV, aged over 60 years, are buyers who frequently want to sell their large dwellings and move into smaller units, which are less expensive and easier to maintain. They are also the potential buyers for the smallest dwellings that they help their children or grandchildren to purchase.

A segmentation of potential green housing buyers in two phases was applied for this study. The first, geographical segmentation, involved the analysis of real estate subdivided into segments of the residential districts under deliberation. The second, demographic (main source of earnings, gender, age, marital status, education, families with children) and customer psychographic and behavioral (happy, angry, valence, sad, and heart rate) segmentation, involved compiling the sum matrices of neuro decision-making for the residential districts under deliberation. The evaluations of the psychographic and behavioral segments of potential housing unit buyers for this research consist of calculated weights of the criteria. The two above-described segmentation phases allow real estate brokers to optimize the available marketing resources by contacting a maximum number of relevant potential customers. Similarly, McGarigal et al. [50] and Strong and Jacobson [51] used a two-step segmentation technique to identify the optimal number of green housing submarkets by considering the characteristics of green housing and attributes of the neighborhoods.

### 3.3. The Sum Segmentation of Four Neighborhoods

In our approach, the first step is the pre-segmentation, wherein a large dataset is grouped into smaller subsegments. In our case, the urban district we were looking at was divided into four neighborhoods (Naujamiesčio, Verkių, Old Town, Žirmūnų). In selecting input variables for our analysis of the sum segmentation of four neighborhoods (Naujamiesčio, Verkių, the Old Town, Žirmūnų), we considered four types of green housing characteristics. Other researchers performed similar studies looking into green housing clusters. Bourassa et al. [52] and Poudyal et al. [53] believe that, in delineating green housing submarkets, four types of green housing characteristics (socioeconomic, structural, neighborhood, and locational) are commonly regarded as the most important factors. To the set of green housing attributes, Jun [54] also added input segment variables such as building age, sales price, floor size, and the number of apartment units in the block; socioeconomic variables such as the average household income and the household head’s education level; and neighborhood and location-related variables such as urban parks, a nearby subway and highway interchanges, and others.

In selecting input variables for the sum segmentation of the four neighborhood (Naujamiesčio, Verkių, Old Town, Žirmūnų) analysis, we considered four types of green housing characteristics:

- individual differences of potential buyers (X_1_–X_6_): age (20–30, 31–40, 41–60 and over 60 years, X_1_), gender (male and female, X_2_), education (higher, high and special secondary, secondary, basic, elementary, incomplete elementary school, X_3_), marital status (married, divorced, widowed, never married, X_4_), seven main source of earnings (X_5_) and families with children (X_6_),
- apartment attributes (X_7_–X_13_): dwelling price (X_7_), type of residential housing unit (X_8_), ownership form of residential dwelling (X_9_), building materials (X_10_), air pollution and noise (X_11_), energy usage (X_12_), and aesthetic attributes (X_13_),
- location impacts (X_14_, X_15_): urban quality (infrastructure) (X_14_) and green spaces (X_15_).
- AVABEPS data (happiness (X_16_), interest (X_17_), valence (X_18_), and arousal (X_19_)) (see Table 6).

### 3.4. Reclassification of the Housing Subsegments from the First Stage Into a Relevant Number of Segments

The second stage involves reclassifying the subsegments from the first stage into a relevant number of segments (see Table 7).

Twenty video advertisements were provided to the group of experts taken from submitted real estate offerings assigned to the corresponding group of potential green housing buyers (see Appendix A Table A1). These data provided the basis for compiling the Old Town aggregated segmentation neuro decision table. All experts denoted the points in the table on their own accord, ranging from 1 to 10 regarding the acquisition/interest potential of a real estate project, considering the requirements along with trends, acquisitions, and stereotypes in the market. Analogous to sum segmentation, neuro decision tables were also compiled for the other Vilnius City districts under analysis (Naujamiestis, Verkiai and Žirmūnai).

Having established the buyer segments in the Old Town, it is possible to perform more relevant marketing to achieve the greatest level of success. The individual differences among buyers, apartment attributes, and location impacts for each buyer segment assist in targeting ads more effectively.

There may be a great deal of possible information that real estate agents could research, use and define, however, the best place to start is with the information that brokers can use in practice. Most importantly, it also contains numerous targeting options that go hand in hand with the buyer segments of brokers. The purpose should always be to understand customers better for more effective communication, as well as to gain the ability to target ads more precisely [55].

As real estate agents learn new information, the buyer segments are likely to change, and, with growing business, brokers may even discover entirely new buyer segments. Defined buyer segments can ensure better ad targeting and communication by brokers. From an increased engagement in marketing, the time taken to define brokers’ buyer segments can help businesses succeed, as this will enable them to know and understand their core customers better [55].

In determining the Old Town neighborhood purchaser segments, we establish with as-broad-as-possible alternatives and then personalized housing toward more concrete potential customers (see Table 7).

Based on the sum neuro decision matrix compiled for the Old Town district during the first-step segmentation, a markedly more personalized neuro decision table is compiled during the second segmentation step (see Table 7). Interested groups can continue to perform the process of dwelling segmentation based on such decision matrices. During such a segmentation process, dwellings may be assembled into similar submarkets relevant to the individual differences of potential buyers (X_1_–X_6_), the attributes of green housing units (X7–X13), location impacts (X14, X15), and AVABEPS data (X_16_–X_19_). By using the COPRAS [35] method and the data from Table 6, the effectiveness of the housing unit video ads in question has been determined. It is obvious that the seventeenth video ad (N_17_ = 100%) is the most effective. N_j_ can vary between 0% and 100%.

## 4. Relation of Human Emotions based on Correlation Analysis between Emotions and Built Environmental State

### 4.1. Built Environment Data for Analysis

Correlations were measured using seven features, describing human emotions (angry, sad, scared, disgusted, and happy), valence, and arousal, with seven features characterizing the built environment (SO_2_, KD_2.5_, KD_10_, NO_2_, CO, O_3_, and Magnetic Storm). As a result, we obtained a matrix of correlations that is given in Table 8. In most cases, the absolute value of correlations exceeds 0.5. There are positive and negative correlations.

The peculiarities of the experiments were as follows:

1. In most cases, the correlations were measured independently of each other.
2. The number of experiments was different for each measurement of correlation.

### 4.2. Multidimensional Scaling for visual Analysis of Correlations

The option to analyze the correlation matrix of human emotions with features characterizing the built environment is Multidimensional Scaling (MDS). This method is the most popular method for a visual representation of multidimensional data in a low-dimensional manner [56,57,58]. It has a number of realizations using artificial neural networks and in combinations with neural networks, too.

In general, when solving real-world data analysis problems, the analyzed objects (items) *X_1_, X_2_,…, X_m_* are characterized by some features *x_1_, x_2_,…, x_n_,* where n is the number of features, and m is the number of objects. The features *x_1_, x_2_,…, x_n_* can achieve some numerical values. A set of these values characterizes a particular object *X_i_* = (*x_i1_, x_i2_,…, x_in_*), *i* ∈ {1,…, *m*}, where i is the order number of the object. If the objects are described by more than one feature, the data characterizing the objects are called multidimensional data. Visual analysis of such data helps gain a deeper insight into the data and to directly interact with the data. In our case, the objects are five human emotions (angry (*X_1_*), sad (*X_3_*), scared (*X_5_*), disgusted (*X_6_*), and happy (*X_7_*)), valence (*X_2_*), and arousal (*X_4_*) (*m = 7*). There are seven features characterizing the built environment (*n = 7*): SO_2_ (*X_1_*), KD_2.5_ (*X_2_*), KD_10_ (*X_3_*), NO_2_ (*X_4_*), CO (*X_5_*), O_3_ (*X_6_*), and Magnetic Storm (*X_7_*).

Low-dimensional visualization requires preserving proximities between objects *X_1_, X_2_,…, X_m_* as much as possible. MDS ensures such an objective, as it is cluster-preserving. MDS requires estimating the coordinates of new points *Y_i_*= (*y_i1_, y_i2_*), *I* = 1,…, *n*, in a lower-dimensional space Rd (*d* = 2) by minimizing some stress function depending on *Y_1_, Y_2_,…, Y_m_*. An example of the stress function may be as follows:

(1) $$E_{MDS}\left(Y\right)=\underset{i<j}{\sum }{\left(d\left(Y_{i},Y_{j}\right)-d\left(X_{i},X_{j}\right)\right)}^{2}$$

Here, *d*(*X_i_, X_j_*) is the proximity between two human emotions, *X_i_, X_j_; Y* = (*Y_1_, Y_2_,…, Y_m_)* is a set of points of lower dimensionality, and (*d<n*); *d* (*Yi, Yj*) is the Euclidean distance between the points, *Y_i_, Y_j_*, in our case. More stress functions are available in Medvedev et al. [57]. The optimization problem is quite complicated because of the number of variables, which is equal to *d×n*, in the general case. Such a large number of variables is determined by the fact that we need to find d coordinates of *n* points. Moreover, the stress function is multimodal, i.e., it has many minima. Usually, gradient-based optimization methods are applied. One of the commonly used algorithms is stress minimization using majorization (SMACOF), which is based on iterative majorization guaranteeing a monotonic convergence. Here, the optimization process is started from initial values of *Y_1_, Y_2_,…, Y_m_.* A particular rule changes these. Finally, such values find that the value of the stress function is as minimal as possible. Two ways are usually applied to the selection of the initial values of *Y_1_, Y_2_,…, Y_m_*. In the simplest way, the values are random numbers from interval (0,1). However, the principal component approach is often used.

The 7×7 matrix of proximities *d* (*Xi, Xj*) between human emotions are presented in Table 9. Elements of the table correspond to the proximity of a particular pair of emotions. We observe high similarities between several pairs of emotions. The proximities of these pairs are tinted in yellow.

### 4.3. Visualization Results and Their Interpretation

**Figure 4:**
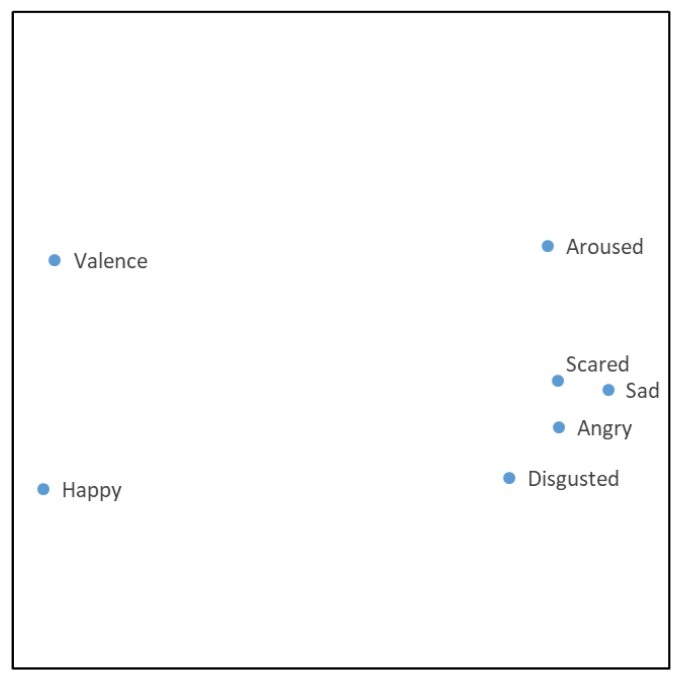
Visualization of a set of human emotions based on built environmental features.

Results of the visualization of features are presented in Figure 4.

Dots in the figures correspond to the emotions. These are named by the corresponding dot. It is possible to evaluate their similarities visually. The more similar emotions appear closer on the plane. The visual presentation on the plane is invariant to the angle of rotation of all points and to the representation’s mirror image. Therefore, the results in Figure 4 may have many interpretations. However, there is no tendency to draw an analogy with the well-known graphical representation of Russell’s circumflex model of emotions for acoustic stimuli [30,59,60]. This is the model where the horizontal axis represents the valence dimension, and the vertical axis represents the arousal dimension.

The main unifying feature of these results is the derivation of formal estimates of similarities of human emotions depending on built environmental features using data science, including big data analysis, visual data analysis and artificial intelligence. The basis for these estimates consists of the influence of the built environment on emotions. Figure 4 displays the different influences of built environmental features on human emotions. There are four clusters of emotions, valence, and arousal:

- Happy
- Valence
- Aroused
- Sad, Scared, Angry, Disgusted

Note that these clusters are obtained based on the proximities of emotions, where the proximities are obtained from the correlation of emotions with built environmental features. Therefore, the built environment influences emotions differently. A greater distance between the points in Figure 4 means a greater specificity of the influence.

The conclusions may be as follows. Russell’s circumplex model of affect [30] proposes that emotions are spread in a two-level round plane, comprising arousal and valence magnitudes. Slight changes in the built environment may lead to emotional changes. The state of the arousal requires more built environmental changes, but not so much when compared with happy and positive valence. Presume the human emotional state is somewhere close to happy or positive valence. The built environment has to change drastically, if the desired emotion (happy) or positive valence has to change to sad, scared, angry, or disgusted. This means it is very difficult to control a positive human emotion when the built environmental features only vary slightly. However, this is possible for the emotions sad, scared, angry, and disgusted.

## 5. Conclusions

Many real estate brokers have noticed that modern buyers are becoming more and more selective. A tremendous amount of effort is required to analyze a tremendous number of variants to arrive at an actual purchase of an apartment.

The performance of a real estate advertisement takes into consideration the individual differences of potential buyers, the desired features of the property, and how the location impacts on the property. These constitute the similarities of this research to prior research studies. However, there was no integrated consideration at the time of the real estate advertising of the physiological, emotional and affective responses of clients and the aforementioned aspects employing a neuro decision matrix. Furthermore, there was no multivariant planning performed on customized, video neuro-advertising variants and multiple criteria analysis. Therefore, the area of the research was expanded. The ViNeRS technique was applied, which includes a combination of physiological, biometric and multiple criteria analysis and multivariant design methods, Damasio’s Somatic Marker Hypothesis, AVABEPS maps, and the employment of a statistical analysis method. The developed ViNeRS Method permits this to be performed. Additionally, this research employed big data (consisting of over 350 million recordings) gained from performing analyses on the arousal and valence, affective behavior, emotional and physiological states of possible buyers of green housing (AVABEPS) in seven intersections of Vilnius City from 7 November 2017 to the point of writing the article in February 2020. Based on this data, over 35,000 strong and average correlations were determined, and they prove the need to use the AVABEPS variables analyzed in this research in neuromarketing. As part of our research, the AVABEPS data were applied in neuromarketing and multicriteria examinations of video advertisements for diverse client segments by applying the neuro decision tables.

The significance of the variable interrelations (see Table 3) and our correlational analysis (see Table 2) support the first hypothesis that “Built environment pollution, including magnetic storms, CO, KD_10_, KD_2.5_, NO_2_, SO_2_, O_3_, influences the emotional states of people (anger, sadness, scared, disgust, and happiness), arousal and valence” (see Section 4). Another point of our findings is that boredom and interest, i.e., human affective reactions, are linked to emotions and physiological states (see Table 4). These relationships have been also confirmed by global findings. Findings by other researchers and the correlational relationships we have identified (see Table 4) support the second hypothesis that “Human affective reactions such as boredom and interest are related with (other) emotional and physiological states”. We have examined the correlational relationships, and our findings show that built environment pollution affects physiological states such as the breathing rate and heart rate. The effect of built environmental pollution on the breathing rate and heart rate of individuals is also evident in global findings. Findings by other researchers and the correlational relationships we have identified (see Table 5) support the third hypothesis that “Physiological states including heart and breathing rates are affected by built environment pollution”. Two case studies were verified and validated. Thereby, the ViNeRS Method was deemed accurate by three of the proposed hypotheses.

The obtained dependencies constituted the basis for calculating and graphically submitting the ViNeRS circumplex model of affect, which the authors of this article had developed. This model is similar to the earlier popular Russell’s circumplex model of affect.

Future research should involve establishing high, medium or low importance for an advertising process by correlating AVABEPS metrics, climatic conditions (air temperature, humidity, average wind velocity, atmospheric pressure, and apparent temperature), and built environment pollution (magnetic storm, SO_2_, KD_2.5_, KD_10_, NO_2_, CO and O_3_). A detailed analysis of the parameter measurements taken in Vilnius with observed strong correlations is needed, because these would greatly affect potential real estate buyers. Rapid decision-making would thus become possible, and problems could be circumvented. The situation at hand offers the best advantage for gain. Furthermore, in the future, we intend to apply various intelligent decision support systems to improve the ViNeRS Method.

## Figures and Tables

**Table 1:** A database snippet with AVABEPS variables of passers-by recorded at four intersections in Vilnius.

Age Groups	Šventaragio and Pilies Sts. intersection		Šventaragio St. and Gedimino Pr. intersection		Kudirkos St. and Gedimino Pr. intersection		Santariškių and Baublio Sts. intersection	
**(a) Happiness**								
	Female	Male	Female	Male	Female	Male	Female	Male
0–20	0.128	0.139	0.128	0.103	0.138	0.163	0.146	0.074
20–30	0.131	0.138	0.125	0.135	0.123	0.154	0.133	0.255
30–40	0.117	0.115	0.116	0.114	0.114	0.101	0.114	0.123
40–50	0.103	0.098	0.081	0.085	0.080	0.089	0.129	0.130
50–60	0.123	0.085	0.089	0.061	0.074	0.066	0.162	0.217
**(b) Heart rate**								
0–20	79,071	76,537	81,791	56,000	69,628	63,667		
20–30	78,779	76,931	75,002	83,914	72,351	70,953		
30–40	75,923	80,393	80,669	72,113	76,228	72,756		
40–50	77,147	74,872	87,742	78,144	86,521	82,111		
50–60	69,539	70,819	97,927	84,312	82,000	83,600		
**(c) Sadness**								
0–20	0.236	0.214	0.213	0.187	0.221	0.170	0.293	0.246
20–30	0.197	0.183	0.195	0.188	0.213	0.168	0.223	0.098
30–40	0.158	0.155	0.161	0.158	0.153	0.129	0.185	0.175
40–50	0.149	0.142	0.176	0.137	0.126	0.116	0.159	0.204
50–60	0.153	0.144	0.108	0.116	0.119	0.098	0.234	0.086
**(d) Anger**								
0–20	0.092	0.116	0.086	0.118	0.087	0.116	0.132	0.116
20–30	0.086	0.122	0.079	0.109	0.103	0.127	0.135	0.074
30–40	0.091	0.119	0.093	0.111	0.089	0.109	0.134	0.157
40–50	0.094	0.123	0.086	0.119	0.094	0.106	0.084	0.126
50–60	0.092	0.129	0.070	0.135	0.071	0.104	0.195	0.106
**(e) Valence**								
0–20	−0.149	−0.134	−0.138	−0.155	−0.128	−0.075	−0.225	−0.220
20–30	−0.102	−0.105	−0.105	−0.109	−0.138	−0.086	−0.158	0.120
30–40	−0.089	−0.102	−0.092	−0.104	−0.093	−0.100	−0.141	−0.147
40–50	−0.107	−0.121	−0.147	−0.131	−0.109	−0.104	−0.225	−0.167
50–60	−0.076	−0.126	−0.081	−0.156	−0.094	−0.106	−0.249	0.001

**Table 2:** AVABEPS and built environment pollution correlational analysis results.

	SO_2_	KD_2.5_	KD_10_	NO_2_	CO	O_3_	Magnetic Storm
Anger	0.489 **	0.507 **	0.306	0.472 **	0.564 **	0.565 **	0.558 **
Valence	−0.613 *	−0.380 *	−0.417 *	−0.498 *	−0.298	−0.621 **	−0.572 **
Sadness	0.740 **	0.511 **	0.515	0.517 **	0.339	0.683 **	0.477 *
Arousal	0.698 *	0.614 **	0.566 **	0.086	0.635 **	0.719 **	−0.170
Scared	0.510	0.510 **	0.605 **	0.501 *	0.550 **	0.571 *	0.402 *
Disgust	0.286	0.181	0.576 **	0.624 **	0.418	0.351 *	0.513 **
Heart rate	0.399 *	0.772 **	0.526 **	−0.353 *	−0.077	0.412	0.590 **
Happiness	−0.788 **	0.695 **	−591 **	−0.217	−0.674 **	−0.673 **	−0.319 *

* The correlation is significant at *p* < 0.05, ** The correlation is significant at *p* < 0.01.

**Table 3:** Significance of links between AVABEPS and built environment pollution variables.

	SO_2_	KD_2.5_	KD_10_	NO_2_	CO	O_3_	Magnetic Storm
Anger	+	+	−	+	+	+	+
Valence	+	+	+	+	−	+	+
Sadness	+	+	−	+	−	+	+
Arousal	+	+	+	−	+	+	−
Scared	−	+	+	+	+	+	+
Disgust	−	−	+	+	−	+	+
Heart rate	+	+	+	+	−	−	+
Happiness	+	+	+	−	+	+	+

**Table 4:** Potential housing buyers’ affective reactions such as boredom and interest and their relation to emotional and physiological states.

	Happy	Angry	Arousal	Sad	Scared	Disgusted	Surprised	Heart Rate	RPM
**Boredom**	−0.951(5)	−0.515(26) −0.530(20) −0.530(20)	−0.751(19) −0.526(19)	−0.565(19) −0.555(19)	−0.504(20) −0.557(6)	−0.522(19)	0.579(20) 0.616(19) 0.769(19) 0.684(19)	−0.658(7) −0.680(6)	0.510(17) 0.516(16)
**Interest**	0.507(20) 0.634(20) 0.724(20)	0.509(13) 0.512(12)	0.885(23) 0.697(19)	−0.564(6)	−0.751(7)	−0.516(15)	0.568(25)	0.505(13) 0.555(12)	0.587(20)

The quantities above are correlations, followed by the number of days examined for the precise correlation specified in parentheses.

**Table 5:** The effect of built environment pollution on physiological states such as the heart rate and breathing rate.

	SO_2_	KD_2.5_	KD_10_	NO_2_	CO	O_3_
RPM	0.817(19) 0.780(19)	0.583(22) 0.521(21)	0.587(14) 0.605(13)	0.601(19) 0.559(19)	0.534(18) 0.650(15)	0.515(134) 0.561(133)
Heart Rate	0.502(12) 0.530(20)	0.782(8) 0.779(7)	0.552(18) 0.530(17)	0.517(26) 0.719(12)	0.591(9) 0.539(9)	0.521(11) 0.564(11)

The quantities above are correlations, followed by the number of days examined for the precise correlation specified in parentheses.

**Table 6:** Sum segmentation, neuro housing decision-making matrix.

Indicators Defining Options	Sub-Indicators Defining Options	Measuring Units	Weight		Housing Unit Video Alternatives Under Comparison				
				*	A_1_	…	A_j_	…	A_n_
**Individual differences of buyers**									
Age (X_1_)	X_11_ (20–30 years)	Points	q _1_	+	x _111_	…	x _11j_	…	x _11n_
	X_12_ (31–40 years)	Points	q _2_	+	x _121_	…	x _12j_	…	x _12n_
	X_13_ (41–60 years)	Points	q _3_	+	X _131_	…	x _13j_	…	x _13n_
	X_14_ (over 60 years)	Points	q _4_	+	x _141_	…	x _14j_	…	x _14n_
Gender (X_2_)	X_21_ (male)	Points	q _5_	+	x _211_	…	x _21j_	…	x _21n_
	X_22_ (female)	Points	q _6_	+	x _221_	…	x _22j_	…	x _22n_
Education (X_3_)	X_31_ (higher)	Points	q _7_	+	x _311_	…	x _31j_	…	x _31n_
	X_32_ (high and special secondary)	Points	q _8_	+	x _321_	…	x _32j_	…	x _32n_
	X_33_ (secondary)	Points	q _9_	+	x _331_	…	X_33j_	…	X_33n_
	X_34_ (basic)	Points	q _10_	+	X_341_	…	x _34j_	…	x _34n_
	X_35_ (elementary)	Points	q _11_	+	X_351_	…	X_35j_	…	x _35n_
	X_36_ (incomplete elementary school)	Points	q _12_	+	X_361_	…	x _36j_	…	x _36n_
Marital status (X_4_)	X_41_ (married)	Points	q _13_	+	X_411_	…	x _41j_	…	x _41n_
	X_42_ (divorced)	Points	q _14_	+	X_421_	…	x _42j_	…	x _42n_
	X_43_ (widowed)	Points	q _15_	+	X_431_	…	x _43j_	…	x _43n_
	X_44_ (never married)	Points	q _16_	+	X_441_	…	x _44j_	…	x _44n_
Main source of earnings (X_5_)	X_51_ (salary/work compensation)	Points	q _17_	+	X_511_	…	x _51j_	…	x _51n_
	X_52_ (income from own or family business)	Points	q _18_	+	X_521_	…	x _52j_	…	x _52n_
	X_53_ (income from agricultural activities)	Points	q _19_	+	X_531_	…	x _53j_	…	x _53n_
	X_54_ (ownership or investment income)	Points	q _20_	+	X_541_	…	x _54j_	…	x _54n_
	X_55_ (pension)	Points	q _21_	+	X_551_	…	x _55j_	…	x _55n_
	X_56_ (governmental support)	Points	q _22_	+	X_561_	…	x _56j_	…	x _56n_
	X_57_ (support by family and/or other persons)	Points	q _23_	+	X_571_	…	x _57j_	…	x _57n_
Families with children (X_6_)	X_61_ (families with children aged 0–17 yrs.)	Points	q _24_	+	X_611_	…	x _61j_	…	x _61n_
	X_62_ (families with no children aged 0–17 yrs.)	Points	q _25_	+	x _621_	…	x _62j_	…	x _62n_
Price (X_7_)	X_71_	Euro per sq. m.	q _26_	-	x _711_	…	x _71j_	…	x _71n_
Type of residential housing unit (X_8_)	X_81_ (one unit house)	Points	q _27_	+	x _811_	…	x _81j_	…	x _81n_
	X_82_ (two-unit house)	Points	q _28_	+	x _821_	…	x _82j_	…	x _82n_
	X_83_ (multi-unit building dwelling)	Points	q _29_	+	x _831_	…	x _83j_	…	x _83n_
Ownership form of residential dwelling (X_9_)	X_91_ (home owner resident)	Points	q _30_	+	x _911_	…	x _91j_	…	x _91n_
	X_92_ (resident in a rental unit)	Points	q _31_	+	x _921_	…	X_92j_	…	x _92n_
Building materials (X_10_)		Points	q _32_	+	x _1011_	…	x _101j_	…	x _101n_
Noise and air pollution (X_11_)		Points	q_33_	+	x _1101_	…	x _110j_	…	x _110n_
Energy consumption (floor heating, renewable energy sources, etc.) (X_12_)		Points	q _34_	+	x _1201_	…	x _120j_	…	x _120n_
Aesthetic features (X_13_)		Points	q _35_	+	x _1301_	…	x _130j_	…	x _130n_
**Environmental influences**									
Urban quality (infrastructure) (X_14_)		Points	q _36_	+	X_1401_	…	X_140 j_	…	X_140 n_
Green spaces (X_15_)		Points	q _37_	+	X_1501_	…	X_150 j_	…	X_150 n_
**AVABEPS data**									
Happiness (X_16_)		Points	q _38_	+	X_1601_	…	X_160 j_	…	X_160 n_
Interest (X_17_)		Points	q_39_	+	X_1701_	…	X_170 j_	…	X_170 n_
Valence (X_18_)		Points	q _40_	+	X_1801_	…	X_180 j_	…	X_180 n_
Arousal (X_19_)		Points	q _41_	+	X_1901_	…	X_190 j_	…	X_190 n_

**Table 7:** Sum neuro housing decision matrix compiled during the Old Town second-stage segmentation.

Indicators Defining Options	Sub-Indicators Defining Options	*	Weight	Measu-ring Units	Housing Unit Video ad Alternatives under Comparison							
					1	2	3		17	18	19	20
**Individual differences of buyers**												
Age	31–40 years	**+**	0.1826	Points	7	6	7	…	5	7	5	6
Gender	Male	**+**	0.1826	Points	7	9	8	…	6	8	6	9
Education	Higher	**+**	0.0925	Points	8	7	7		7	8	7	8
Marital status	Married	**+**	0.0989	Points	7	6	7		8	9	8	9
Main source of earnings	Salary/work compensation	**+**	0.1398	Points	7	8	7		4	7	5	7
Families with children	Families with no children	**+**	0.0560	Points	8	8	8		8	7	8	7
**Apartment-style unit attributes**												
Price (G1)	Average price (1 euro sq. m.)	**–**	0.8	€/m^2^	1830	1450	1700		4050	1290	3710	1320
Type of residential housing unit	Multi-unit building dwelling	**+**	0.1662	Points	8	9	8		7	9	7	9
Ownership form of residential dwelling	Resident in a rental unit	**+**	0.0730	Points	7	8	8		5	8	5	7
Building materials		**+**	0.096	Points	7	5	7		9	6	9	7
Noise and air pollution		**+**	0.08	Points	8	8	7		8	8	8	7
Energy usage		**+**	0.184	Points	6	7	7		8	9	9	7
Aesthetic properties		**+**	0.04	Points	7	7	8		9	8	9	7
**Environmental influences**												
Urban quality (infrastructure)		**+**	0.144	Points	7	7	8		9	6	9	7
Green spaces		**+**	0.096	Points	7	7	7		6	7	7	6
**AVABEPS data**												
Happiness		**+**	0.1	Points	0.135	0.135	0.135		0.135	0.135	0.135	0.135
Interest		**+**	0.1	Points	0.013	0.013	0.013		0.013	0.013	0.013	0.013
Valence		**+**	0.1	Points	−0.131	−0.131	−0.131		−0.131	−0.131	−0.131	−0.131
Arousal		**+**	0.1	Points	0.330	0.330	0.330		0.330	0.330	0.330	0.330
Significance *Q_j_* of housing unit video alternatives					0.0845	0.0887	0.0853		0.1057	0.0926	0.1005	0.0917
Priority of housing unit video alternatives					18	10	14		1	6	2	8
Utility degree *N_j_* of housing unit video alternatives (%)					79.99	83.92	80.76		100	87.59	95.07	86.78

*—The + (−) specifies that either a greater or lower criterion value means greater (lower) significance for customers.

**Table 8:** Correlation matrix of potential housing buyers’ emotions with features characterizing the built environment.

	SO_2_	KD_2.5_	KD_10_	NO_2_	CO	O_3_	Magnetic Storm
Angry	0.489	0.507	0.306	0.472	0.564	0.565	0.558
Valence	−0.613	−0.380	−0.417	−0.498	−0.298	−0.621	−0.572
Sad	0.740	0.511	0.515	0.517	0.339	0.683	0.477
Arousal	0.698	0.614	0.566	0.086	0.635	0.719	−0.170
Scared	0.510	0.510	0.605	0.501	0.550	0.571	0.402
Disgusted	0.286	0.181	0.576	0.624	0.418	0.351	0.513
Happy	−0.788	0.695	−0.591	−0.217	−0.674	−0.673	−0.319

**Table 9:** Table of proximities of potential housing buyers’ emotions, valence, and arousal for Multidimensional Scaling.

	Angry	Valence	Sad	Arousal	Scared	Disgusted	Happy
Angry	0.000	2.625	0.424	0.911	0.340	0.559	2.604
Valence	2.625	0.000	2.780	2.616	2.680	2.450	1.226
Sad	0.424	2.780	0.000	0.842	0.352	0.669	2.762
Arousal	0.911	2.616	0.842	0.000	0.759	1.138	2.705
Scared	0.340	2.680	0.352	0.759	0.000	0.503	2.689
Disgusted	0.559	2.450	0.669	1.138	0.503	0.000	2.534
Happy	2.604	1.226	2.762	2.705	2.689	2.534	0.000

## Appendix A

**Table A1:** Video advertisements on housing units and their descriptions.

Line No.	Name of RE Project	Video, Tour, Gallery	RE Project Descriptions	Targeted Age Group *
1.	Ozo kvartetas (apartment-style dwelling units)	http://ozokvartetas.lt/virtualus-turas/	Small, economy class apartment-style units designated for a young, modern person without a family. New buildings surround this project. It has an average distance from the city center. The entire, required infrastructure is nearby.	I, IV
2.	Ozo parkas (apartment-style dwelling units)	http://ozoparkas.lt/galerija/ozo-parkas/	This project is economy to middle class. Buyers are offered apartment-style, 1 to 3-room dwelling units. This is an entire residential micro-region with new buildings. A recreational zone is alongside the park. The target audience consists of young families; however, persons of various ages are accommodated.	II, IV
3.	Miesto ritmu (apartment-style dwelling units in the Šnipiškės district)	https://www.miestoritmu.lt/lt/galerija	Average class project located in the city’s central district. Offers to buyers are for apartment-style, 1 to 4-room dwelling units. A center of numerous city businesses neighbors the project. The target group of buyers consists of employed persons. Excellent accessibility with the city’s historical center as well as with the other districts is available.	II, III
4.	Žirmūnų skveras (apartment-style dwelling units in the Žirmūnai district)	http://zirmunuskveras.lt/galerija/	Average class project is in a central district of the city. Offers to buyers are for apartment-style, 1 to 4-room dwelling units. Excellent accessibility with the city’s historical center as well as with the other districts is available. A river runs along the project. Pedestrian walkways including a recreational zone are available.	II, III
5.	Raitininkų sodai (apartment-style dwelling units in the Žirmūnai district)	https://www.yit.lt/bustas/nauji-butai-vilniuje/raitininku-sodas	Upper average class project in the city’s center accommodates families with small children along with older persons. The construction quality is high. Excellent accessibility with the city’s historical center as well as with the other districts is available. A river runs along the project. Pedestrian walkways including a recreational zone are available.	II, III
6.	Namų pynės (apartment-style dwelling units in the Žirmūnai district)	http://www.namupynes.lt/galerija	Average class project is in a central district of the city. Offers to buyers are for apartment-style, 1 to 3-room dwelling units. Excellent accessibility with the city’s historical center as well as with the other districts is available. A river runs along the project. Pedestrian walkways including a recreational zone are available.	II, III
7.	Rinktinės namai (apartment-style dwelling units in the Verkiai district)	https://www.youtube.com/watch?v=eFpwwkm9Zd4(video-filmas)	Average class project is in a central district of the city. Offers to buyers are for apartment-style, 2 to 4-room dwelling units. Excellent accessibility with the city’s historical center as well as with the other districts is available. Many business centers of the city neighbor the project. The target buyer group consists of employed persons.	II, III
8.	Veikmės parko namai (apartment-style dwelling units in the Baltupiai district)	http://www.veikme.lt/uploads/files/dir248/dir12/8_0.php	A modern average class project in one of the bedroom districts. Nature surrounds the project. It displays exceptional architectural and engineering decisions that employ passive home technologies.	II, III
9.	Levandų namai (apartment-style dwelling units in the Pašilaičiai district)	https://www.youtube.com/watch?v=sfQMUE6A5F4(video-filmas)	Economy class project is in one of the more distant districts of the city. Offers of small apartment-style, dwelling units are for young families or older single people.	I, II
10.	Elguvos deimantai (apartment-style dwelling units in the Karoliniškės-Ozas district)	https://www.youtube.com/watch?v=UpcKkYncjVo(video-filmas)	Elguvos Deimantai is a low-rise, construction project located in the Karoliniškės district. It merges with its neighboring woodlands. Fencing encompasses the entire project. Its construction materials are high quality containing heat insulation. It contains underground and ground parking and storage sheds. Potential buyers include older people, generally businesspeople and high-level specialists.	II, III
11.	Kalvarijų St. multi-unit building dwelling	https://www.youtube.com/watch?v=AI_scq37sFA(video-filmas)	Apartment-style dwelling units in a residential micro-region are located near the city’s center. The units are part of a monolithic building of an old construction that has been remodeled. Potential buyers are single persons or families of average and lower incomes.	I, IV
12.	Pavasaris (apartment-style dwelling units in the Lazdynai district)	https://www.youtube.com/watch?v=7FxqyynUjDo(video-filmas)	Economy class project in one of the city’s bedroom residential districts is at a distance from the city’s center. Still, it assures the entire, needed infrastructure and full services. It neighbors a recreational zone. Potential buyers include families satisfied to live at a distance from the city’s center along with existing residents of the district.	II, IV
13.	Žirgų 1 (houses, cottages)	http://www.zirgu1.lt/360/; http://www.zirgu1.lt/gyvenviete-2/	This is an economy class square of cottages located in the Vilnius suburbs. It is a price alternative dwelling, the same as many other cottages in this class. Its location is at a distance from the city’s center. Potential buyers include families desiring greater spaciousness for whom the distance from the city’s center has no relevance.	II, III
14.	Valakampių krantas (cottages)	http://valakampiukrantas.lt/?gclid=EAIaIQobChMI3PXHlOmA3gIVBhUYCh1vJg45EAMYASAAEgLpWPD_BwE	Luxury class project built with high quality buildings and modern engineering decisions. The neighborhood is solid and stable. Nature surrounds the project. It is for those who desire a comfortable life style but have no need to be near the city’s center. It suits persons in higher income and older groups.	III
15.	Baltupio krantas (cottages)	https://www.youtube.com/watch?v=cVIhfHqvfSc(video-filmas); http://baltupiokrantas.lt/	Luxury class project is located in a bedroom district at about an average distance from the city’s center. It suits buyers who desire greater privacy, spaciousness and nature but without sacrificing ease of accessibility with the city’s center. It is suitable for older people.	II, III
16.	Brick house in the Kalnėnai district	https://www.youtube.com/watch?v=NE3aMYbmS24(video-filmas)	Brick house constructed in 2004 in the Kalnėnai district, which is under intensive development in Vilnius. This is a fully equipped, spacious house with a garage and other subsidiary facilities. It has a convenient driveway. It suits families; though, the social infrastructure is still not fully developed (it has no nurseries, shops or the like).	II, III
17.	Live square	http://livesquare.lt/galerija-vizualizacijos/	Luxury class project located in the city’s center. It is equally as suitable as an investment or for a modern, comfortable residence. The highest standards in construction quality are assured. Excellent accessibility in all directions combines with an excellent infrastructure and solid neighborhood. Potential buyers consist of older people, generally businesspeople or high-level specialists.	II, III
18.	Karaliaučiaus slėnis	https://www.karaliauciausslenis.lt/galerija/181?c=v	Economy class cottages harmoniously constructed in a square, located in a bedroom district. This is an alternative to an apartment-style unit by price, the same as are many other cottages of this class. However, it is at a distance from the city’s center. This project was awarded for harmonious development in 2016. Potential buyers include families requiring greater space for whom the city’s center is not a relevant aspect.	II, III
19.	CNTRL	http://www.cntrl.lt/apie-projekta/48	Luxury class project located in the city’s center is as suitable for an investment as for a modern, convenient residence. The highest standard in construction quality is assured. Accessibility is excellent in all directions. The infrastructure is excellent and the neighborhood, solid. Potential buyers include older people, generally businesspeople or high-level specialists.	III
20.	Vilniaus vakarai	http://vilniausvakarai.lt/butai-kotedzai/	Economy class apartment-style units and cottages in a bedroom district are available in an area with an undeveloped infrastructure. Thus potential buyers must be mobile. It suits those who search from a natural setting and who want relaxation from the city’s hubbub.	II, III

* Target age groups: I—(20–30) II—(31–40) III—(41–60) IV—(60+).
